# Reduction of environmental distraction to facilitate cognitive performance

**DOI:** 10.3389/fpsyg.2014.00860

**Published:** 2014-08-06

**Authors:** Annelies Vredeveldt, Timothy J. Perfect

**Affiliations:** ^1^Department of Criminal Law and Criminology, VU University AmsterdamAmsterdam, Netherlands; ^2^School of Psychology, University of PlymouthPlymouth, UK

**Keywords:** distraction, cognitive load, modality-specific interference, eye-closure, eyewitness memory

## Introduction

Imagine explaining a statistics problem to a student while your colleague at the back of the room is frantically waving to get your attention. Or imagine reporting to a police officer on the street what happened during a witnessed mugging, while seeing traffic buzz by and hearing snippets of conversations from passers-by. Environmental distractions can have an impact on cognitive performance, whether this concerns solving a mathematical problem, maintaining a conversation, or retrieving an experienced event from memory. Glenberg et al. (1998) were the first to systematically explore the relationship between memory, environmental distraction, and behavioral control of distraction through gaze aversion and eye-closure. In a series of experiments, they found that people are more likely to avert their gaze when trying to answer more difficult questions about general and autobiographical information. Moreover, they found that instructed eye-closure resulted in better performance on a word recall task, whereas watching a silent movie resulted in poorer performance. Inspired by this work, Wagstaff et al. (2004) and Perfect et al. (2008) examined whether instructed eye-closure could also improve recall of events. In a series of studies, they found that eye-closure substantially improved the amount and accuracy of information reported about witnessed events.

## Theoretical issues

Different mechanisms have been proposed to explain the eye-closure effect, which can be divided into two broad categories: general versus modality-specific. The general-distraction explanation is based on Glenberg's (1997) embodied cognition account of memory, which holds that environmental monitoring and cognitive tasks such as memory retrieval compete for cognitive resources. Disengaging from the environment (e.g., through eye-closure) allows us to reallocate cognitive resources to the task at hand, thus improving performance, but at the potential cost of poorer monitoring of the current environment. The general-distraction explanation of the eye-closure effect is supported by findings that, in some studies, eye-closure improved recall of both visual and auditory information (e.g., Perfect et al., 2008, Experiments 4 and 5; Vredeveldt and Penrod, 2013, free recall). It is also supported by the finding that eye-closure can reduce the cross-modal memory impairment caused by auditory distraction (Perfect et al., 2011). The modality-specific explanation, on the other hand, holds that distractions in the environment only interfere with concurrent tasks in the same modality, consistent with Baddeley and Hitch's (1974) working memory model (see also Baddeley and Andrade, 2000). This explanation is supported by findings that, in some studies, eye-closure improved recall only for visual details (e.g., Perfect et al., 2008, Experiment 2; Vredeveldt et al., 2012; Vredeveldt and Penrod, 2013, cued recall). Further, recall of visual details is most disrupted by visual distraction, whereas recall of auditory details is most disrupted by auditory distraction (Vredeveldt et al., 2011). All in all, it seems likely that both general and modality-specific processes are involved in the effect of environmental distraction on cognitive performance.

Although the role of modality has received much research attention, other aspects of the nature of the distraction are relatively less well-investigated. In this Research Topic, issues such as the social aspects of environmental distractions (Buchanan et al., 2014), and the relevance of the distraction to pending goals (Scheiter et al., 2014) and other tasks (Weeks and Hasher, 2014) are further explored. In addition, different aspects of performance are addressed, such as response criterion (Rae and Perfect, 2014) and other metacognitive indices (Beaman et al., 2014). We also learn more about the neural basis of the effect of distraction on performance (Wais and Gazzaley, 2014).

## Applied issues

Research on the effect of distraction on cognitive performance has clear practical implications. In educational settings, students must remember large quantities of information to perform well on examinations. In medical settings, doctors often rely on patients' memory reports to establish medical histories and identify appropriate treatment options. In legal settings, information provided by eyewitnesses plays a pivotal role in police investigations and legal decisions. With respect to the latter, many interviewing procedures have been developed to help witnesses remember more, and some have been found to be highly successful, such as the Cognitive Interview (Fisher and Geiselman, 1992) and the NICHD protocol (Orbach et al., 2000). However, practical implementation of such complex protocols has proven difficult (e.g., Clarke and Milne, 2001). The Eye-Closure Interview (Vredeveldt et al., submitted) could prove to be a feasible and effective alternative. Indeed, findings from studies in which the eye-closure instruction was tested under naturalistic conditions (Vredeveldt and Penrod, 2013) and in a field setting (Vredeveldt et al., submitted) seem promising.

Several contributions in the Research Topic specifically focused on practical applications. For example, Scheiter et al. (2014) examined how distraction affects students' learning in educational hypermedia environments. Hyman et al. (2014) explored the impact of talking on the phone on walking behavior, an all-too-common form of distraction that exemplifies the trade-off between attention to the internal and external worlds discussed by Glenberg (1997). Additionally, two articles investigated the effectiveness of reducing distraction through eye-closure in interviews with child witnesses (Kyriakidou et al., 2014; Mastroberardino and Vredeveldt, 2014).

## Conclusion

The aims of this Research Topic were two-fold: (1) to enhance our understanding of the mechanisms involved in the effects of distraction on cognitive performance, and (2) to identify methods that successfully reduce environmental distractions, thus facilitating cognitive performance in applied settings. The articles present state-of-the-art research providing novel insights into these key questions, and Craik's (2014) commentary constitutes an excellent critical review of this important work. In all, the contributions in this Research Topic advance our knowledge of both theoretical and applied aspects of the effects of environmental distraction on cognitive performance. Understanding how distraction affects performance, and how we can effectively reduce the impact of distraction, could prove fruitful in improving cognitive performance in a wide range of applied settings. Procedures such as eye-closure or noise reduction may assist students to concentrate on their exams, help witnesses to remember more about criminal events, and could improve the reader's chances of winning their next pub quiz, though whether they would enjoy a distraction-free pub environment is another matter.

### Conflict of interest statement

The authors declare that the research was conducted in the absence of any commercial or financial relationships that could be construed as a potential conflict of interest.
